# Prenatal Exposure to Air Pollution and Immune Thrombocytopenia: A Nationwide Population-Based Cohort Study

**DOI:** 10.3389/fped.2022.837101

**Published:** 2022-03-18

**Authors:** Hsin-Chien Yen, Chien-Heng Lin, Ming-Chih Lin, Ya-Chi Hsu, Yi-Hsuan Lin

**Affiliations:** ^1^Children’s Medical Center, Taichung Veterans General Hospital, Taichung City, Taiwan; ^2^Department of Medical Research, Taichung Veterans General Hospital, Taichung City, Taiwan; ^3^School of Medicine, National Yang Ming Chiao Tung University, Taipei City, Taiwan; ^4^Department of Food and Nutrition, Providence University, Taichung City, Taiwan; ^5^School of Medicine, Chung Shan Medical University, Taichung City, Taiwan; ^6^Department of Post-baccalaureate Medicine, College of Medicine, National Chung Hsing University, Taichung City, Taiwan

**Keywords:** immune thrombocytopenia (ITP), air pollution, prenatal exposure, children, pollutant standard index (PSI), PM10, PM10 (particulate matter)

## Abstract

**Introduction:**

Immune thrombocytopenia (ITP) is one of the most common hematologic disorders in children. However, its etiology is still unclear. Epidemiological studies have shown that air pollution is a plausible risk factor in stimulation of oxidative stress, induction of inflammation, and onset of autoimmune diseases. The objective of this article is to examine the effects of prenatal exposure to air pollution on the occurrence of immune thrombocytopenia (ITP) in children.

**Materials and Methods:**

This is a nationwide, population-based, matched case-control study. Using data from Taiwan’s Maternal and Child Health Database (MCHD), we identified 427 children with ITP less than 6 years of age and age-matched controls without ITP between 2004 and 2016. Levels of prenatal exposure to air pollutants were obtained from 71 Environmental Protection Administration monitoring stations across Taiwan according to the maternal residence during pregnancy. Patients who had outpatient visits or admission with diagnosis of ITP and subsequently received first-line treatment of intravenous immunoglobulin or oral glucocorticoids were defined as incidence cases.

**Results:**

Prenatal exposure to particulate matter <10 μm (PM10) in diameter and the pollutant standard index (PSI) increased the risk of childhood ITP. Conversely, carbon monoxide (CO) exposure during pregnancy was negatively associated with the development of ITP.

**Conclusion:**

Certain prenatal air pollutant exposure may increase the incidence of ITP in children.

## Introduction

Immune thrombocytopenia (ITP) is one of the most common hematologic disorders in children, which is characterized by isolated thrombocytopenia (platelet count <100,000/uL with normal hemoglobin level and white blood cell count) and presents with an increased bleeding risk. Children with ITP can present at any age, but it occurs mainly in young children; 50 percent of the patients were between one and 4 years of age and approximately 80 percent were younger than 8 years of age (1–3). The pathogenesis of ITP remains incompletely understood. It might be triggered by a viral infection or other immunologic triggers (4). Aberrant immune response to some environmental triggers in individuals with genetic susceptibility may also contribute to the pathogenesis of ITP (5).

Air pollution is reported to be associated with stimulation of oxidative stress, induction of inflammation and onset of autoimmune diseases (6, 7). Prenatal air pollution exposure, as an intrauterine toxin and a strong oxidant, is associated with poor pregnancy outcomes and long-term health effects in the offsprings (8). Epidemiological studies have also revealed prenatal exposure to air pollution as a risk factor for small for gestational age, preterm birth, impaired newborn lung, impaired immune function, brain developmental disorders, and cognitive disorders after birth (9–12). Children’s acquired immune disorders, such as allergic diseases and Kawasaki disease, have also been reported to be associated with prenatal air pollution exposure (13–16). However, data regarding prenatal exposure to air pollution and its possible association with ITP are lacking. This nationwide longitudinal study assessed the correlation between prenatal and early-life air pollution exposure to the development of ITP.

## Materials and Methods

### Study Design and Data Source

This is a nationwide, population-based, matched case-control study. The data source was Taiwan’s Maternal and Child Health Database (MCHD), which is governed by the Health and Welfare Data Science Center (HWDC) of the Ministry of Health and Welfare (MOHW). The MCHD consists of Taiwan’s birth registry, birth notifications, mortality registrations, and the National Health Insurance Research Database (NHIRD) (17). The National Health Insurance (NHI) program was launched in Taiwan in March 1995, and includes comprehensive medical claims data from 99% of Taiwanese population of nearly 24 million (18, 19). The National Health Insurance Research Database (NHIRD) comprises all-inclusive claims data on patient’s registration, residence, demographic characteristics, examinations, diagnoses, procedures, medical expenditure, surgeries, medication prescriptions, inpatient services, and outpatient services (20). By integrating these databases, we obtained a detailed linked profile of all births to mothers from the MCHD, and we extracted both inpatient and outpatient medical claims data of children and mothers from the NHIRD. The main data sources for analysis were the inpatient expenditures by admission (DD) and the ambulatory care expenditures by visit (CD) files. Diagnoses in the NHIRD are coded using the International Classification of Diseases, Ninth Revision, Clinical Modification (ICD-9-CM) format. To protect patients’ identity and validate the reliability of the databases, investigators are demanded to perform onsite analysis at HWDC *via* remote connection to MOHW servers (21). This study protocol was approved by the institutional review board of Taichung Veterans General Hospital (CE17178A-4). The review board waived the requirement for written informed consent as all patient data were anonymized prior to analysis.

### Case Identification

The cohort included births from 2004 to 2010. Births with unclear information on residence, multiple delivery, brothers or sisters (only the first child during the study period in a family was included), or congenital anomaly (ICD-9 code 740 to 758) were excluded from the analysis. Children under 6 years old with a diagnosis of ITP (ICD-9 code 287.3 and ICD-10 code D69.3) who were treated with either intravenous immunoglobulin (IVIG, ATC code: J06BA02) or oral glucocorticoids, including dexamethasone (ATC code: H02AB02) or prednisolone (ATC code: H02AB06) between January 2004 to December 2016 were identified as cases (22). The control groups were matched to the ITP group by age and index month, and were randomly chosen at a control-to-case ratio of 4:1. The cohort was followed up until the end of 2016 to ensure that every child in this cohort was followed up for at least 6 years.

### Assessment of Exposure to Air Pollutants

Concentrations of each air pollutant, including Sulfur dioxide (SO_2_), Ozone (O_3_), Carbon monoxide (CO), Carbon dioxide (CO_2_), particulate matter <10 μm in diameter (PM10), particulate matter <2.5 μm in diameter (PM2.5), Nitric Oxide (NO), Nitrogen dioxide (NO_2_), and Nitrogen Oxides (NO_x_), have been measured hourly by 76 Environmental Protection Administration monitoring stations across Taiwan since 1999 (23). Among them, 5 national park and background air quality monitoring stations were excluded from the analysis. We estimated the air pollutant levels at each residential location utilizing a spatio-temporal model established *via* a deep-learning approach (24). The Pollutant Standards Index (PSI), a type of air quality index, was determined by the highest value for the five pollutants (i.e., SO_2_, PM_10_, NO_2_, CO, and O_3_) after transforming their concentrations into a scale from 0 through 500 (25). By using the analysis of Cressman, we computed the PSI and the average concentrations of these air pollutants in 368 townships across Taiwan (26). Finally, we apportioned the prenatal air pollutant exposures in each township to the child according to their mother’s residential postal code during pregnancy.

### Statistics

Data were expressed as either frequency and percentage or mean and standard deviation. Continuous variables were compared using the *t*-test, and categorical variables were compared using the Pearson’s chi-square test. We selected covariates based on data availability, expert knowledge, and the literature. The association between prenatal exposure to air pollution and risk of ITP was examined using a multiple logistic regression analysis after adjusting for age, gender, maternal age, mode of delivery, birth weight, maternal illness, and allergic disease. We also calculated the odds of ITP per interquartile range (IQR) change in PSI level to determine the dose-dependent effect. All data were analyzed using the SAS statistical package (version 9.4; SAS Institute, Cary, NC, United States). A *p* < 0.05 was considered statistically significant.

## Results

### Baseline Characteristics of Immune Thrombocytopenia and Age-Matched Controls

**FIGURE 1:**
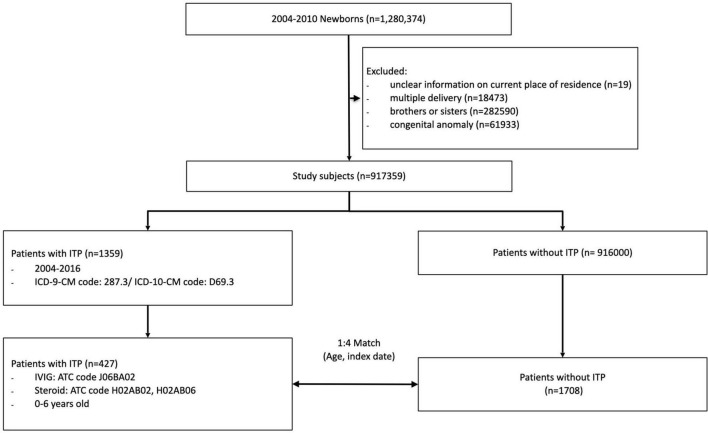
Composition of the study cohort.

Of the 917359 children born in Taiwan from 2004 through 2010, 427 children under the age of 6 years old with ITP were identified as the ITP group. A total of 1,708 children (1:4) without ITP, matched by age and index date, were randomly selected as the control group (Figure 1). Patients in the ITP group and the those in non-ITP group had similar distributions of age, birth weight, maternal age, mode of delivery, gestational age, maternal comorbidity, and allergic disease. ITP occurred mostly at the age of 0–2 years, with a significant male predominance (60.9%) (Table 1).

**TABLE 1:** Baseline characteristics of ITP and control groups.

Characteristics	Non-ITP group (*n* = 1,708)	ITP group (*n* = 427)	*p*-value
	*n (%)*	*n (%)*	
Neonatal age (years)			1.000
0–1	456 (26.7)	114 (26.7)	
1–2	392 (23)	98 (23)	
2–3	216 (12.6)	54 (12.6)	
3–4	180 (10.5)	45 (10.5)	
4–5	228 (13.3)	57 (13.3)	
5–6	236 (13.8)	59 (13.8)	
Neonatal gender			0.001
Female	820 (48)	167 (39.1)	
Male	888 (52)	260 (60.9)	
Birth weight (g)			0.882
≥2,500	1623 (95)	405 (94.8)	
<2,500	85 (5)	22 (5.2)	
Maternal age			0.723
<35	14,85(86.9)	374 (87.6)	
≥35	223 (13.1)	53 (12.4)	
Mode of delivery			0.295
Vaginal delivery	1110 (65)	289 (67.7)	
Cesarean section	598 (35)	138 (32.3)	
**Maternal comorbidity**			
Diabetes mellitus	10 (0.6)	4 (0.9)	0.421
Hypertension	10 (0.6)	3 (0.7)	0.781
Preterm delivery			0.301
≥37 weeks	1607 (94.1)	396 (92.7)	
<37 weeks	101 (5.9)	31 (7.3)	
**Allergic disease**			
Asthma	46 (2.7)	17 (4)	0.160
Allergic rhinitis	316 (18.5)	66 (15.5)	0.142
Atopic dermatitis	43 (2.5)	8 (1.9)	0.436

### Prenatal Exposure to Air Pollutants

When comparing air pollutant exposure between ITP and non-ITP groups, levels of prenatal exposure to PSI, and PM_10_ were significantly higher in patients with ITP than in the matched controls. On the other hand, exposure to SO_2_, CO_2_, O_3_, PM2.5, NOx, NO, and NO_2_ did not show any significant differences. Interestingly, exposure to CO was found to have an inverse association with the risk of ITP development (Table 2).

**TABLE 2:** Prenatal exposure to air pollutants in ITP group and matched control group.

Air pollutants	Non-ITP group (*n* = 16,768)	ITP group (*n* = 4,192)	*p-*value
	Mean ± SD	Mean ± SD	
PSI	57.13 ± 7.24	58 ± 7.92	0.041
SO_2_ (ppb)	4.46 ± 1.55	4.51 ± 1.52	0.60
CO (ppm)	0.53 ± 0.11	0.51 ± 0.11	0.027
CO_2_ (ppm)	884.57 ± 702.23	886.1 ± 913.27	0.98
O_3_ (ppb)	28.46 ± 2.87	28.66 ± 3	0.19
PM10 (μg/m^3^)	58.99 ± 13.82	60.59 ± 13.92	0.032
PM2.5 (μg/m^3^)	57.24 ± 82.63	53.7 ± 56.4	0.31
NOx (ppb)	25.46 ± 6.71	24.8 ± 6.91	0.07
NO (ppb)	6.37 ± 2.57	6.15 ± 2.64	0.11
NO_2_ (ppb)	19.1 ± 4.4	18.66 ± 4.54	0.06

*PSI, Pollutant Standards Index; SO_2_, Sulfur dioxide; CO, carbon monoxide; CO_2_, carbon dioxide; O_3_, ozone; PM2.5, particulate matter ≦2.5 μm; PM10, particulate matter ≦10 μm in diameter; NO, nitric oxide; NO_2_, nitric dioxide; NO_x_, nitrogen oxide.*

### Multiple Logistic Regression Analysis of Factors Associated With Immune Thrombocytopenia

A multiple logistic regression model was applied to analyze the associations between exposure to each air pollutant (i.e., SO_2_, CO, CO_2_, O_3_, PM_2_._5_, PM_10_, NO, NO_2_, and NO_*x*_) or PSI and ITP. After adjustment for potential confounders (age, gender, maternal age, mode of delivery, preterm, maternal comorbidity, and allergic disease), prenatal air pollutant exposure calculated by PSI was demonstrated to be associated with a significantly higher risk for developing ITP in early childhood. On the other hand, prenatal CO exposure showed a protective effect (Table 3).

**TABLE 3:** Multivariate analysis of factors associated with ITP^a^.

Characteristics	Air pollution exposure during pregnancy		
	OR	95%CI	*p*-value
PSI	1.016	1.001–1.031	0.032
SO_2_ (per 10 ppb)	1.001	0.995–1.008	0.67
CO (per 10 ppm)	0.900	0.815–0.993	0.037
CO_2_ (per 10 ppm)	1.000	1.000–1.000	0.82
O_3_ (per 10 ppb)	1.003	0.999–1.006	0.16
PM_10_ (per 10 μg/m^3^)	1.001	1.000–1.002	0.039
PM_2_._5_ (per 10 μg/m^3^)	1.000	1.000–1.000	0.39
NO_*x*_ (per 10 ppb)	0.999	0.997–1.000	0.08
NO (per 10 ppb)	0.997	0.992–1.001	0.12
NO_2_ (per 10 ppb)	0.998	0.995–1.000	0.08

*^a^Model adjusted for Age, Gender, Birth weight, Mother age, Mode of delivery, Preterm delivery, Maternal comorbidity and allergic disease. CI, confidence interval; CO, carbon monoxide; CO_2_, carbon dioxide; NO, nitric oxide; NO_2_, nitric dioxide NO_x_, nitrogen oxide; O_3_, ozone; OR, odds ratio; PM2.5, particulate matter 2.5 μM; PM10, particulate matter 10 μM; PSI, Pollutant Standards Index; SO_2_, Sulfur dioxide.*

### Dose-Dependent Effect of Air Pollution on Risk of Immune Thrombocytopenia

**FIGURE 2:**
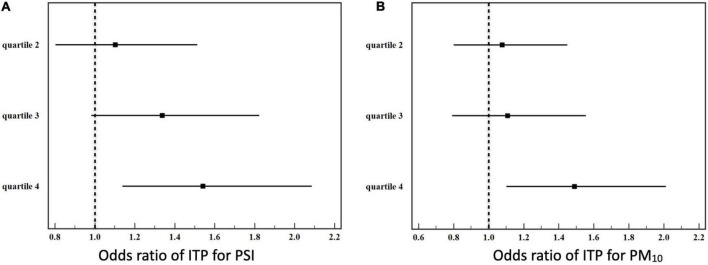
Quartiles of prenatal cumulative air pollutant exposure to the risk of ITP. **(A)** Pollutant standard index (PSI), **(B)** PM_10_.

Quartiles of the cumulative amounts of air pollutants were then calculated to analyze the dose- dependent effect of prenatal exposure on ITP. The odds ratios of ITP increased with increasing in cumulative amounts of pollutants. Linear trends could be observed for PSI and PM_10_ (Figure 2).

## Discussion

This study investigated the association between prenatal exposure to air pollution and the development of pediatric ITP. We observed prenatal exposure to certain types of air pollutants increased the risk of IPP in early childhood. The study is the first investigation assessing the effect of prenatal exposure to air pollutants on childhood ITP. Furthermore, this study is a nationwide, population-based cohort study.

Immune thrombocytopenia, an autoimmune disease, is characterized by bleeding episodes, purpura, and isolated thrombocytopenia resulted from antiplatelet autoantibodies (3). The predominant pathophysiological theory for ITP is an antibody-mediated destruction against targeted platelets leading to a markedly reduced platelet lifespan and defective megakaryopoiesis (3). Among ITP patients, abundant surface antigens on platelet membrane, such as glycoprotein (GP) Ib/IX, GP IIb/IIIa, GP Ia/IIa, and GP VI can be binded by autoantibodies (27). Both genetic and acquired factors contribute to the pathogenesis of ITP. However it hasn’t been completely understood. Among the environmental factors, the proinflammatory effects of air pollutants have been reported to be associated with the generation of autoimmunity and inflammation (28). As a strong oxidant and an intrauterine toxin, prenatal exposure to particulate matter was associated with long-term complications in the offspring, including impaired neonatal lung function, immune function disorders, and abnormal organogenesis (8). Ambient air pollution has been suspected to trigger autoimmune disease (29). Epidemiological studies have provided authentic evidence of the relationship between air pollution and subsequent occurrence of autoimmune diseases in human, such as diabetes mellitus type 1, juvenile idiopathic arthritis, systemic sclerosis, systemic lupus erythematosus (SLE), autoimmune myositis, and rheumatic arthritis (28, 30–33). Prenatal air pollutants exposure has also been reported to be associated with the development of Kawasaki disease, childhood asthma, and atopic dermatitis (13, 34). Those evidences support our hypothesis that prenatal exposure to air pollutants can prompt postnatal immune dysregulation, which eventually results in increased risk of pediatric ITP.

Laboratory studies also provide evidence to support that prenatal particulate matter exposure to be associated with intrauterine inflammation by revealing higher maternal C-reactive protein levels among exposure mothers (35, 36). In addition, animal models also reveal the link between antenatal inflammation and an alteration in subsequent postnatal immune response (37, 38). Moreover, prenatal exposure to air pollution has been reported to elevate fetal C-reactive protein levels and to influence lymphocyte immunophenotypes and interleukin-1b production (35, 39, 40). Furthermore, epigenetic studies have also shown that air pollution can alter prenatal and postnatal organ development (41, 42). Epigenetic contribution to autoimmune disease has also been reported (43).

Interestingly, a negative association of CO exposure during pregnancy with ITP in children was observed. CO is commonly identified as a poisonous gas because of its better affinity to hemoglobin than oxygen and formation of carboxyhemoglobin, leading to the tissue hypoxia due to impaired oxygen-carrying capacity in circulation (44). Nevertheless, many studies have revealed its cytoprotective properties and the anti-inflammatory effect of CO through regulating the balance between anti-inflammatory Th2 cells and pro-inflammatory Th1/Th17 (44). Given that CO serves as a potential therapeutic bioactive molecule, the therapeutic potential has been reported in experimental models, including ischemia-reperfusion injury, (45) hypoxia, (46) and autoimmune diseases (47). Indeed, these studies have recognized CO as a potential pharmaceutical agent for autoimmune diseases for its anti-apoptotic and anti-inflammatory effects.

There are some limitations in this study. First, it is possible that some pediatric patients with ITP did not seek medical advice as no symptoms or only mild symptoms were experienced, and cases of ITP might have been underestimated in the NHIRD. However, it is reasonable to assume that the majority of patients who did not seek medical help had less severe disease activity. Indeed, children with moderate to severe disease burden are of greater concern. Second, we apportioned township-level air pollution exposure to each case and did not calculate or measure individual exposure, including height of residence or proximity to major roads, which could have resulted in misclassification.

In summary, our results demonstrated that prenatal exposure to air pollution may be associated with the development of ITP in preschool-aged children. More research is needed to elucidate the causal relationships between maternal exposure to air pollutants and development of ITP in early childhood.

## Data Availability Statement

The data analyzed in this study is subject to the following licenses/restrictions: To protect patients’ identity and validate the reliability of the databases, investigators are required to perform onsite analysis at HWDC *via* remote connection to MOHW servers. Requests to access these datasets should be directed to C-HL, epid@ms39.hinet.net.

## Ethics Statement

The studies involving human participants were reviewed and approved by Institutional review board of Taichung Veterans General Hospital. Written informed consent from the participants’ legal guardian/next of kin was not required to participate in this study in accordance with the national legislation and the institutional requirements.

## Author Contributions

H-CY wrote the manuscript. C-HL performed data anslysis. M-CL designed the research and review the final version of the manuscript. Y-CH helped in data analysis. Y-HL helped in the study design. All authors contributed to the article and approved the submitted version.

## Conflict of Interest

The authors declare that the research was conducted in the absence of any commercial or financial relationships that could be construed as a potential conflict of interest.

## Publisher’s Note

All claims expressed in this article are solely those of the authors and do not necessarily represent those of their affiliated organizations, or those of the publisher, the editors and the reviewers. Any product that may be evaluated in this article, or claim that may be made by its manufacturer, is not guaranteed or endorsed by the publisher.
